# Examining the Impact of Question Construction on Reporting of Sexual Identity: Survey Experiment Among Young Adults

**DOI:** 10.2196/32294

**Published:** 2021-12-13

**Authors:** William J Young, Michelle T Bover Manderski, Ollie Ganz, Cristine D Delnevo, Mary Hrywna

**Affiliations:** 1 Center for Tobacco Studies Rutgers Biomedical and Health Sciences Rutgers University New Brunswick, NJ United States; 2 Department of Biostatistics and Epidemiology Rutgers School of Public Health Rutgers University Piscataway, NJ United States; 3 Department of Health Behavior, Society and Policy Rutgers School of Public Health Rutgers University Piscataway, NJ United States

**Keywords:** survey measurement, sexual identity, survey wording experiment

## Abstract

**Background:**

Compared with heterosexuals, sexual minorities in the United States experience a higher incidence of negative physical and mental health outcomes. However, a variety of measurement challenges limit researchers’ ability to conduct meaningful survey research to understand these disparities. Despite the prevalence of additional identities, many national health surveys only offer respondents 3 substantive options for reporting their sexual identities (straight/heterosexual, gay or lesbian, and bisexual), which could lead to measurement error via misreporting and item nonresponse.

**Objective:**

This study compared the traditional 3-option approach to measuring sexual identity with an expanded approach that offered respondents 5 additional options.

**Methods:**

An online survey experiment conducted among New Jersey residents between March and June 2021 randomly assigned 1254 young adults (ages 18-21) to answer either the 3-response measure of sexual identity or the expanded item. Response distributions for each measure were compared as were the odds of item nonresponse.

**Results:**

The expanded version of the question appeared to result in more accurate reporting among some subgroups and induced less item nonresponse; 12% (77/642) of respondents in the expanded version selected a response that was not available in the shorter version. Females answering the expanded item were less likely to identify as gay or lesbian (2.1% [10/467] vs. 6.6% [30/457]). Females and Non-Hispanic Whites were slightly more likely to skip the shorter version than the longer version (1.1% [5/457 for females and 3/264 for Non-Hispanic Whites] vs. 0% [0/467 for females and 0/277 for Non-Hispanic Whites]). About 5% (32/642) of respondents answering the longer item were unsure of their sexual identity (a similar option was not available in the shorter version). Compared with respondents answering the longer version of the question, those answering the shorter version had substantially greater odds of skipping the question altogether (odds ratio 9.57, 95% CI 1.21-75.74; *P*=.03).

**Conclusions:**

Results favor the use of a longer, more detailed approach to measuring sexual identity in epidemiological research. Such a measure will likely allow researchers to produce more accurate estimates of health behaviors and outcomes among sexual minorities.

## Introduction

Compared with heterosexual individuals, those identifying as sexual minorities in the United States experience a higher incidence of negative physical and mental health outcomes [1-7]. They also report higher levels of risk behaviors including tobacco, alcohol, and drug use [1,2,8-13]. Given that sexual minorities bear a disproportionate burden of risk behaviors and poor health outcomes, research to understand and address these health inequities is essential [14]. However, survey measurement challenges limit the ability to conduct meaningful research inclusive of sexual minorities. Indeed, a variety of approaches to operationalizing sexual orientation exist across national surveys, complicating estimates of risk behaviors and health outcomes among this population [15,16]. In fact, the National Academies of Sciences, Engineering, and Medicine commissioned a panel to review current measures and methodological issues related to measuring sexual orientation, in addition to sex and gender identity [17].

In this short paper, we contribute to the literature on measuring sexual identity by presenting the results of a randomized experiment comparing 2 measurement approaches. It is well established in the literature on survey methods that question design can affect respondents’ motivation to respond accurately, or even at all, to particular items [18]. If a question does not motivate respondents to answer accurately, or it encourages them to skip the item altogether (eg, item nonresponse), this can lead to measurement error [18]. One common approach to measure sexual identity asks participants to choose from 1 of 3 responses: heterosexual/straight, gay or lesbian, and bisexual. This 3-response approach, or a close variation of it, is the one taken by several national surveys, including the National Health Interview Survey [19], Behavioral Risk Factor Surveillance System [20], and the National Survey on Drug Use and Health [21]. Despite the popularity of this approach, these 3 responses do not constitute an exhaustive list of sexual identities that one may claim [22]. In failing to offer a broader range of options, surveys employing the 3-response approach are susceptible to measurement error, either because respondents report an inaccurate sexual identity or because they skip the item altogether if they believe it does not represent their actual identities.

## Methods

To explore the impact of question construction on measurement of sexual identity, we randomly assigned a diverse group of 1254 young adults, aged 18-21 years, to answer either the traditional, 3-response version of the sexual identity item (n=612) or an expanded version offering more response options (n=642). Overall sample demographics and demographics by experimental condition are presented in Multimedia Appendix 1. The traditional, 3-category question read, “Do you consider yourself to be:” and offered 3 response options: “Heterosexual or straight,” “Gay or lesbian,” and “Bisexual.” The longer version read, “Below is a list of terms that people often use to describe their sexuality or sexual orientation. Please select the term that best applies to you.” It offered the responses, “Straight/Heterosexual,” “Gay,” “Lesbian,” “Bisexual,” “Queer,” “Asexual,” “Pansexual,” “Questioning/Not sure,” and “Another sexual orientation not listed above (please specify).” The experiment was embedded in the first wave survey of the Policy and Communication Evaluation: New Jersey (PACE NJ) study. The survey was fielded online between March 24 and June 21, 2021. In addition to the age requirement, participants in the PACE NJ study were required to report living in New Jersey for at least four months out of the year.

## Results

The expanded version of the question offers a more complete picture of respondents’ sexual identities (Tables 1 and 2). In fact, 12% (77/642) of respondents answering the longer question selected a response option that was not offered in the shorter, more commonly used version of the question. Cross-tabular results revealed that the proportion of females identifying as gay or lesbian was much lower in the expanded version compared with the shorter version (2.1% [10/467] vs. 6.6% [30/457]), as they presumably opted for terms such as queer (4.1% [19/467]) or pansexual (2.6% [12/467]). Females and Non-Hispanic Whites were slightly more likely to skip the shorter version than the longer version (1.1% [5/457 for females and 3/264 for non-Hispanic Whites] vs. 0% [0/467 for females and 0/277 for Non-Hispanic Whites]). Importantly, 6.6% (31/467) of females, 6.1% (22/359) of non-White respondents, and 5% (32/642) overall reported questioning or being unsure of their sexuality in the expanded version. It could be, then, that some individuals avoided answering the shorter item not only because they felt the choices did not represent their identities, but also because they were unsure of their identities in the first place.

**Table 1:** Response distributions by sex and race in experimental condition 1.^a^

Sexual identity		Overall (N=612)		Sex						Race				
				Male (n=155)		Female (n=457)			Non-White (n=339)				White, non-Hispanic (n=264)	
		n (%)	95% CI	n (%)	95% CI	n (%)	95% CI	n (%)			95% CI	n (%)		95% CI
**Condition 1: Do you consider yourself to be:**														
	Heterosexual or straight	426 (69.6)	66 to 73	125 (80.6)	74 to 87	301 (65.9)	62 to 70	254 (74.9)			70 to 80	165 (62.5)		57 to 68
	Gay or lesbian	43 (7.0)	5 to 9	13 (8.4)	4 to 13	30 (6.6)	4 to 9	17 (5.0)			3 to 7	26 (9.8)		6 to 13
	Bisexual	134 (21.9)	19 to 25	13 (8.4)	4 to 13	121 (26.5)	22 to 31	63 (18.6)			14 to 23	70 (26.5)		21 to 32
	Queer	N/A	N/A	N/A	N/A	N/A	N/A	N/A			N/A	N/A		N/A
	Asexual	N/A	N/A	N/A	N/A	N/A	N/A	N/A			N/A	N/A		N/A
	Pansexual	N/A	N/A	N/A	N/A	N/A	N/A	N/A			N/A	N/A		N/A
	Questioning/not sure	N/A	N/A	N/A	N/A	N/A	N/A	N/A			N/A	N/A		N/A
	Other (specify)	N/A	N/A	N/A	N/A	N/A	N/A	N/A			N/A	N/A		N/A
	Missing	9 (1.5)	0.5 to 2	4 (2.6)	0.1 to 5	5 (1.1)	0.1 to 2	5 (1.5)			0.2 to 3	3 (1.1)		0 to 2

^a^Percentages may not add to 100 due to rounding; race categories do not add to overall totals due to missing data.

^b^N/A: not applicable.

**Table 2:** Response distributions by sex and race in experimental condition 2.^a^

Sexual identity			Overall (N=642)				Sex										Race							
							Male (n=175)					Female (n=467)					Non-White (n=359)					White, non-Hispanic (n=277)		
			n (%)		95% CI		n (%)		95% CI		n (%)			95% CI		n (%)			95% CI		n (%)			95% CI
**Condition 2: Below is a list of terms that people often use to describe their sexuality or sexual orientation. Please select the term that best applies to you.**																								
	Heterosexual or straight	423 (65.9)		62 to 70		133 (76.0)		70 to 82		290 (62.1)			58 to 67		250 (69.6)			65 to 74		170 (61.4)			56 to 67	
	Gay or lesbian	30 (4.7)		3 to 6		20 (11.4)		7 to 16		10 (2.1)			0.8 to 3		10 (2.8)			1 to 5		19 (6.9)			4 to 10	
	Bisexual	111 (17.3)		14 to 20		15 (8.6)		4 to 13		96 (20.6)			17 to 24		51 (14.2)			11 to 18		59 (21.3)			16 to 26	
	Queer	20 (3.1)		2 to 4		1 (0.6)		0 to 2		19 (4.1)			2 to 6		9 (2.5)			0.8 to 4		11 (4.0)			2 to 6	
	Asexual	5 (0.8)		0.1 to 2		1 (0.6)		0 to 2		4 (0.9)			0.04 to 2		3 (0.8)			0 to 2		2 (0.7)			0 to 2	
	Pansexual	15 (2.3)		1 to 3		3 (1.7)		0 to 4		12 (2.6)			1 to 4		10 (2.8)			1 to 5		5 (1.8)			0.2 to 3	
	Questioning/not sure	32 (5.0)		3 to 7		1 (0.6)		0 to 2		31 (6.6)			4 to 9		22 (6.1)			4 to 9		9 (3.2)			1 to 5	
	Other (specify)	5 (0.8)		0.1 to 2		0 (0)		0 to 0		5 (1.1)			0.2 to 2		3 (0.8)			0 to 2		2 (0.7)			0 to 2	
	Missing	1 (0.2)		0 to 0.5		1 (0.6)		0 to 2		0 (0)			0 to 0		1 (0.3)			0 to 0.9		0 (0)			0 to 0	

^a^Percentages may not add to 100 due to rounding; race categories do not add to overall totals due to missing data.

An additional indicator of question performance is participants’ willingness to respond to the question they received. As noted above, if some respondents felt that the shorter version of the question did not well represent their actual sexual identities, or if they were unsure of their identities, then we should expect to see a greater propensity toward item nonresponse than in the longer, more complete version of the question. To test this hypothesis, we estimated a logistic regression in which item nonresponse was regressed on a dummy treatment variable. Indeed, compared with respondents answering the longer version of the question, those answering the shorter version had substantially greater odds of skipping the question altogether (odds ratio 9.57, 95% CI 1.21-75.74; *P*=.03). Additionally, this has important implications for survey design: if survey questions are used as screeners and branch to additional items based on the sexual identity measure, then the magnitude of the impact of item nonresponse will increase.

## Discussion

Considered together, the comparison of response distributions (Tables 1 and 2) and the analysis of respondents’ willingness to answer the question they received cast doubt on the appropriateness of the shorter, 3-category approach to measuring sexual identity. The longer item presents a descriptively richer picture of respondents’ identities and induced significantly lower odds of item nonresponse. Furthermore, if the limited, shorter survey question makes respondents feel excluded, it could result in further stigmatizing or marginalizing individuals with nonnormative sexual identities [23]. Given that sexual minorities are more likely to experience negative health outcomes and report higher levels of some risk behaviors, these findings warrant attention from those aiming to study such outcomes and accurately describe their prevalence among various groups in the United States [1]. This is especially so given that sexual minorities are not a homogenous group in terms of health outcomes [1].

This study has limitations. Given that respondents were randomized between question versions, the internal validity of the study is high. However, this sample consisted only of young adults between the ages of 18 and 21. Further research should explore whether the impact of receiving one question version over another varies by respondent age. Moreover, our experimental respondents all live at least four months of the year in New Jersey. If comfort levels with revealing information about one’s sexual identity vary regionally, the sizes of the treatment effects presented here may also vary if this experiment were fielded in other parts of the country or nationally.

To conclude, the evidence presented here favors the use of a longer, more detailed approach to measuring sexual identity in epidemiological research. This measure will likely allow researchers to produce more accurate estimates of health behaviors and outcomes among sexual minorities, given that respondents are less likely to skip the question, compared with the shorter item. Furthermore, accounting for the fluidity of sexual identity in the survey question will help to improve inclusion and representation in survey research among sexual minorities [23].

## Appendix

Multimedia Appendix 1 Sample Demographics.

## Abbreviations

FDA: Food and Drug Administration
NCI: National Cancer Institute
PACE NJ: Policy and Communication Evaluation: New Jersey
