# Supplementary material for: Examining the Impact of Question Construction on Reporting of Sexual Identity: Survey Experiment Among Young Adults
Source: JMIR Public Health Surveill. 2021 Dec 13;7(12):e32294. doi: 10.2196/32294 (PMC8713107; doi:10.2196/32294)
Supplement: Multimedia Appendix 1 [file publichealth_v7i12e32294_app1.docx]

|  | **Overall** | **Condition 1** | **Condition 2** |
| --- | --- | --- | --- |
|  | %(N) | %(N) | %(N) |
| **Age** |  |  |  |
| 18 | 20 (248) | 21 (126) | 19 (122) |
| 19 | 27 (344) | 29 (175) | 26 (169) |
| 20 | 29 (363) | 28 (174) | 29 (189) |
| 21 | 24 (299) | 22 (137) | 25 (162) |
| **Sex** |  |  |  |
| Male | 26 (330) | 25 (155) | 27 (175) |
| Female | 74 (924) | 75 (457) | 73 (467) |
| **Hispanic** |  |  |  |
| Yes | 17 (215) | 18 (109) | 17 (106) |
| **Race**^a^ |  |  |  |
| American Indian/Alaska Native | 2 (22) | 2 (10) | 2 (12) |
| Asian | 32 (401) | 31 (187) | 34 (214) |
| Black/African American | 11 (141) | 12 (71) | 11 (70) |
| Native Hawaiian/other Pacific Islander | 1 (11) | 1 (5) | 1 (6) |
| White | 54 (667) | 55 (329) | 53 (338) |
| Other | 7 (86) | 7 (43) | 7 (43) |
| Missing | 1 (15) | 2 (9) | 1 (6) |
| **Education** |  |  |  |
| No formal education | 0 (2) | 0 (1) | 0 (1) |
| Less than high school | 3 (37) | 4 (23) | 2 (14) |
| High school/GED | 31 (384) | 30 (186) | 31 (198) |
| Some college | 57 (719) | 56 (345) | 58 (374) |
| Associate’s/trade school | 5 (63) | 5 (30) | 5 (33) |
| Bachelor’s degree | 4 (49) | 4 (27) | 3 (22) |
| Total N= | 1254 | 612 | 642 |
| ^a^Note: Overall race categories add to 1343: respondents could select multiple | | | |
